# A virtual recruitment protocol promotes enrollment of underrepresented groups in a diabetes prevention trial

**DOI:** 10.1017/cts.2024.11

**Published:** 2024-02-05

**Authors:** Natalie D. Ritchie, Melanie T. Turk, Jodi Summers Holtrop, Michael Josh Durfee, L. Miriam Dickinson, Peter G. Kaufmann

**Affiliations:** 1 Center for Health Systems Research, Denver Health and Hospital Authority, Denver, CO, USA; 2 Department of Psychiatry, University of Colorado School of Medicine, Aurora, CO, USA; 3 Duquesne University School of Nursing, Pittsburgh, PA, USA; 4 Adult & Child Center for Outcomes Research & Delivery Science, University of Colorado Anschutz Medical Campus, Aurora, CO, USA; 5 Department of Family Medicine, University of Colorado Anschutz Medical Campus, Aurora, CO, USA; 6 Integrated Health Sciences, University of Nevada Las Vegas, Las Vegas, NV, USA

**Keywords:** Clinical trials, health equity, recruitment, technology, social determinants of health

## Abstract

Strategies are needed to ensure greater participation of underrepresented groups in diabetes research. We examined the impact of a remote study protocol on enrollment in diabetes research, specifically the Pre-NDPP clinical trial. Recruitment was conducted among 2807 diverse patients in a safety-net healthcare system. Results indicated three-fold greater odds of enrolling in remote versus in-person protocols (AOR 2.90; *P* < 0.001 [95% CI 2.29–3.67]). Priority populations with significantly higher enrollment included Latinx and Black individuals, Spanish speakers, and individuals who had Medicaid or were uninsured. A remote study design may promote overall recruitment into clinical trials, while effectively supporting enrollment of underrepresented groups.

## Introduction

Ensuring that diverse populations participate in clinical trials is essential to understanding how people from different backgrounds respond to interventions. Recruiting diverse populations is especially important in research to prevent and manage diabetes, which has a disparately high prevalence among racial and ethnic minority groups, older adults, and individuals of low socioeconomic status [1]. However, there is often limited inclusion of underrepresented groups in diabetes trials. A 2022 review found that 62.3% of diabetes trials inadequately recruited Asian, Black, and Hispanic participants, relative to their share of the US population [2]. Various recommendations have been made to enroll more underrepresented groups in clinical trials by addressing social determinants of health (SDOH), including community engagement, employing culturally- and demographically-matched research staff, establishing trust, providing informational sessions about the trial, and offering sufficient compensation for transportation costs and time [3–5]. Nonetheless, such approaches to improving the recruitment of underrepresented groups often fall short [6], and additional strategies are needed.

The COVID-19 pandemic led to rapidly expanding uses of technology to conduct diabetes care and research following remote protocols [7]. This event presented a unique opportunity to assess the extent to which, and for whom, remote protocols may improve outcomes of interest, including enrollments in clinical trials. Thus far, reports show that diverse and predominately low-income populations may prefer virtually-delivered diabetes interventions [8] and may have improved outcomes [9]. However, there are concerns about whether virtual recruitment methods can bridge the “digital divide” or may exacerbate inequities in diverse inclusion by omitting individuals with limited financial means, English proficiency, and/or availability of technology and internet access [10,11]. The COVID-19 pandemic provided an opportunity for us to examine whether remote protocols can support the enrollment of diverse participants, rather than resulting in over-enrollment of economically-advantaged, technologically-savvy participants. This brief report describes the impact of a remote protocol on the enrollment of underrepresented groups in diabetes research, specifically the Pre-NDPP clinical trial [12].

## Methods

The Pre-NDPP study is a large randomized controlled trial (RCT) to assess the effects of a motivational “pre-session” that is added to standard delivery of the National Diabetes Prevention Program (NDPP) [12]. The target population is a diverse and predominately low-income population. The Pre-NDPP protocol was successfully piloted in a prior observational study [13] and merited rigorous research in an RCT. The Pre-NDPP trial was conducted at Denver Health, which is a safety-net healthcare system with the 6^th^ largest network of Federally Qualified Health Centers in the US. Eligible participants included English- and Spanish-speaking adults with a body mass index (BMI) ≥25 kg/m^2^ (≥23 kg/m^2^ if Asian race) and either prediabetes (e.g., A1C 5.7%–6.4%), past gestational diabetes, or an elevated score on a risk questionnaire (https://www.cdc.gov/diabetes/prevention/pdf/prediabetestest.pdf). Potential participants were identified through provider- and self-referrals, and a risk registry based on medical record data.

Before the COVID-19 pandemic, from July 2019 to March 2020, we implemented an in-person protocol [12]. Study visits and intervention delivery were conducted onsite, involving face-to-face interaction with research staff. Given the planned enrollment of ∼25% Spanish speakers and ∼67% Latinx participants, most staff members (*n* = 4 of 5) were bilingual and bicultural. We offered transportation assistance as needed. Recruitment was halted for four months due to the pandemic. From August 2020 to January 2023, we resumed recruitment using a remote protocol for all research activities. Enrollment procedures and criteria were identical for both in-person and remote protocols. First, study staff screened medical records to confirm initial eligibility for potential participants. Potential participants were then reached by phone to gauge interest and schedule the baseline study visit. Initial outreach was also conducted through mail, e-mail, and text messages. At the baseline visit, consenting participants were randomized to the Pre-NDPP or standard NDPP arms of the study. To conduct research activities remotely, we used phone- and video-conferencing, e-consenting, and electronic surveys. We provided body weight scales and instructed participants to text or e-mail a picture of the scale reading to confirm their current weight (weight change is the primary outcome). The Colorado Multiple Institutional Review Board (18-2542) approved all study modifications.

### Analyses

The subpopulations were categorized from medical record data on sex (female or male), age (18–44; 45–64; or ≥65 years); race and ethnicity (Latinx; Non-Latinx Black; Non-Latinx white; or Other); primary language (Spanish or English), insurance (Medicaid/Uninsured, Medicare only, or private insurance); and BMI (25–29.9 or ≥30 kg/m^2^). The characteristics of all outreached individuals were compared with chi-square tests to assess differences between those who were offered the in-person or remote protocol. Logistic regression models assessed the likelihood of enrollment with the remote protocol, compared to the in-person protocol, among all outreached participants and within subpopulations. Adjusted models controlled for the other respective subpopulation characteristics. For example, adjusted models that predicted enrollment among older adults controlled for sex, race and ethnicity, language, insurance, and BMI. We also controlled for the initial identification method (provider-referred, self-referred, or no referral), initial contact method (phone or e-mail/text message/mail), and which staff member conducted the outreach (three of whom recruited with both the in-person and remote protocols, one staff member who recruited in-person only, and one who recruited remotely only). The goal was detecting differences in enrollment success with the in-person vs. remote study protocol, rather than other potential factors that could influence enrollment [14].

As relative normalcy in the US resumed by 2022 [15], a sensitivity analysis compared the likelihood of enrolling with the remote protocol between January 2022 and January 2023, and all previous enrollments with the in-person protocol. Thus, we may limit potential confounding of the pandemic on remote enrollments. That is, during the initial waves of COVID-19 pandemic, participants may have been more inclined to enroll remotely, given fewer alternatives and competing demands because of stay-at-home orders, unemployment, etc.

## Results

Table 1 shows the characteristics of all 2807 individuals who were outreached for enrollment in the Pre-NDPP trial, including 1528 and 1279 individuals who were outreached with the in-person and remote protocols, respectively. Most outreached individuals were female (67.5%), <65 years old (91.9%), from racial and ethnic minority groups (84.5%), English-speaking (60.5%), had Medicaid or were uninsured (84.7%), and had obesity (71.5%). Potential participants who were outreached with either the in-person or remote protocol were similar in terms of their sex, race and ethnicity, primary language, insurance, and BMI. There were relatively more adults ages 45–64 and ≥65 years (and fewer adults <45 years) who were outreached in the remote protocol than with the in-person protocol.

**Table 1.** Characteristics of all outreached participants in the pre-NDPP trial with in-person vs. remote protocols (*N* = 2807)

Characteristic	In-person protocol before COVID (*n* = 1528)		Remote protocol after COVID (*n* = 1279)		
	*n*	%	*n*	%	*P*-value
Sex					
Female	1032	67.5%	864	67.6%	0.970
Male	496	32.5%	414	32.4%	–
Age (years)					
18–44	751	49.2%	523	41.2%	**<0.001**
45–64	667	43.7%	626	49.4%	**0.004**
≥65	109	7.1%	119	9.4%	**0.035**
Race & Ethnicity					
Latinx	1090	71.8%	879	70.1%	0.337
Non-latinx Black	154	10.1%	142	11.3%	0.314
Non-latinx white	238	15.7%	192	15.3%	0.796
Other	37	2.4%	41	3.3%	0.186
Primary language					
Spanish	577	37.9%	521	41.4%	0.054
English	947	62.1%	736	58.6%	–
Insurance					
Medicaid or uninsured	1302	86.0%	1075	86.1%	0.957
Medicare only	36	2.4%	30	2.4%	0.967
Private insurance	176	11.6%	144	11.5%	0.938
Body mass index (kg/m^2^)					
25–29.9	443	29.4%	338	27.3%	0.217
≥30	1063	70.6%	901	72.7%	–

Data are presented as the frequency of study sample characteristics and p-values for chi-square tests of differences between the in-person and remote protocols. Other race and ethnicity includes Asian and Pacific Islander (*n* = 33), American Indian and Alaska Native (*n* = 13), Latinx Black (*n* = 9), and Other Not Hispanic, Latinx, or Spanish Origin (*n* = 23). Bold text indicates *P* < 0.05.

In adjusted models, individuals who were outreached with the remote study protocol were nearly three times more likely to enroll than those who were outreached with the in-person protocol (AOR 2.90; *P* < 0.001 [95% CI 2.29–3.67]). Table 2 shows adjusted odds of study enrollment with the remote vs. in-person protocol for each subpopulation. Among traditionally underrepresented groups, there were significantly greater odds of enrolling in the remote protocol (compared to the in-person protocol) for Latinx and non-Latinx Black individuals, Spanish speakers, and individuals who had Medicaid or were uninsured. Other groups with significantly greater odds of enrolling in the remote protocol (compared to the in-person protocol) were females, adults <45 years, adults 45–64 years, English speakers, and patients with overweight or obesity.

**Table 2.** Likelihood of enrollment in the pre-NDPP trial with remote study protocol compared to the in-person protocol (*N* = 2807)

Subpopulation	In-person protocol before COVID		Remote protocol after COVID		Likelihood of enrollment in Remote vs. In-person protocol	
	Enrolled *n*/ Outreached *n*	%	Enrolled *n*/ Outreached *n*	%	AOR (95% CI)	*P*-value
Sex						
Females	99/1032	9.6%	282/864	32.6%	3.47 (2.64–4.57)	**<0.001**
Males	38/496	7.7%	63/414	15.2%	1.60 (0.99–2.59)	0.054
Age (years)						
18–44	54/751	7.2%	158/523	29.7%	3.88 (2.70–5.56)	**<0.001**
45–64	71/667	10.6%	162/626	25.9%	2.41 (1.71–3.40)	**<0.001**
≥65	12/109	11.0%	26/119	21.8%	1.90 (0.76–4.74)	0.170
Race & Ethnicity						
Latinx	94/1090	8.6%	255/879	29.0%	3.22 (2.42–4.26)	**<0.001**
Non-latinx Black	15/154	9.7%	37/142	26.1%	2.27 (1.09–4.75)	**0.029**
Non-latinx white	25 /238	10.5%	40/192	20.8%	1.72 (0.93–3.20)	0.086
Other	3/37	8.1%	9/41	22.0%	4.30 (0.72–25.66)	0.110
Primary language						
Spanish	53/577	9.2%	157/521	30.1%	3.06 (2.07–4.52)	**<0.001**
English	84/947	8.9%	184/736	25.0%	2.85 (2.10–3.86)	**<0.001**
Insurance						
Medicaid or uninsured	117/1302	9.0%	301/1075	28.0%	3.05 (2.36–3.93)	**<0.001**
Medicare only	5/36	13.9%	5/30	16.7%	0.46 (0.04–4.71)	0.510
Private insurance	14/176	8.0%	33/144	22.9%	2.17 (0.98–4.77)	0.055
Body mass index (kg/m^2^)						
25–29.9	35/443	7.9%	90/338	26.6%	3.72 (2.29–6.05)	**<0.001**
≥30	97/1063	9.1%	248/901	27.5%	2.79 (2.12–3.68)	**<0.001**
Total	137/1528	9.0%	346/1279	27.1%	2.90 (2.29–3.67)	**<0.001**

Data are presented as the frequency and adjusted odds ratio for enrolling in the remote study protocol compared to the in-person protocol. In-person enrollment is the reference group. Models controlled for the other respective subpopulation characteristics, the way that a potential participant was initially identified (provider-referred, self-referred, or no referral), how a potential participant was contacted (phone or e-mail/text message/mail), and which staff member conducted the outreach activities. AOR = Adjusted odds ratio with 95% confidence interval. Bold text indicates *P* < 0.05.

Results from sensitivity analyses were fully consistent with the adjusted models. The unadjusted results were also consistent, but with all groups appearing to favor enrollment in the remote protocol. The unadjusted models reached significance for males (OR 2.16; *P* < 0.001 [95% CI 1.41–3.31]); older adults ≥65 years (OR 2.26; *P* = 0.031 [95% CI 1.08–4.74]); individuals with private insurance (OR 3.44; *P* < 0.001 [95% CI 1.76–6.72]); and non-Latinx white individuals (OR 2.24; *P* = 0.003 [95% CI 1.30–3.85]).

### Discussion

A remote study protocol appears to be well-accepted by a diverse and predominately low-income population with diabetes risks in a clinical trial of the NDPP. The remote study protocol led to about 25% enrollment among outreached individuals, compared to about 10% enrollment with the in-person protocol. Moreover, there were notable gains in enrollment among Latinx, Black, and low-income individuals when the study protocol was offered remotely. Employing a remote study design may support overall recruitment into clinical trials, while effectively supporting the enrollment of underrepresented groups.

Our findings align with a recent qualitative study that describes how participants preferred remote protocols for outreach (especially e-mail and telephone communication), providing consent, and participating in research during the COVID-19 pandemic [16]. In contrast, remote NDPPs have shown disparately low recruitment of racial and ethnic minority groups [17]. Our findings may assuage concerns that remote programs only benefit those with consistent access to technology, or necessary insurance benefits [17]. Rather, one unique contribution of this study is demonstrating that a remote protocol successfully enrolled priority populations in diabetes research. Another important finding is that groups with overweight/obesity were 3-4 times more likely to enroll with the remote than in-person protocol, consistent with previous findings about enrollment trends in a digital DPP [18]. A possible explanation is that a remote setting may be more comfortable and feel less stigmatizing for individuals with overweight/obesity. Moreover, remote participation imparts fewer logistic and time challenges that may be particularly burdensome for underserved populations.

Despite overall gains in enrollment with the remote protocol, our results suggest that remotely conducted trials may need targeted recruitment efforts to enrich study samples with males and older adults. For example, approximately two female participants enrolled for every male with our remote study protocol, which would lead to imbalance. Although another concern is that older adults did not show a greater preference for enrolling in the remote protocol (their enrollment nearly doubled but the difference was not statistically significant after accounting for other factors). However, a recent study revealed substantial gains in technology use among older adults over the past decade [19]. As of 2021, 75% of older adults are internet users and 61% own a smartphone, up from only 13% of older adults owning a smartphone in 2012 [19]. If trends continue, remote protocols may be increasingly favorable to older adults.

Possible explanations for the study findings are that groups facing the greatest barriers to research participation may most benefit once those barriers are removed. Indeed, a UK study also showed relatively high odds of completing a digital DPP among racial and ethnic minority participants [18]. Additionally, retired older adults may have enough leisure time to devote to in-person activities, whereas younger adults may be especially incentivized to engage in remote activities that do not conflict with their competing demands.

Limitations include using insurance as a proxy for income and lacking more complete measures of SDOH (e.g., housing stability, food insecurity, employment status) [20]. Our data also come from one trial conducted in a single healthcare system. Further study in other research centers, including trials with different population segments and disease conditions, is likely needed to corroborate results and increase generalizability. The findings may also be impacted by the COVID-19 pandemic, including how potential participants may have been extra-motivated to address diabetes risks that were associated with poor COVID-19 outcomes. Given the success of remote enrollment, we did not resume in-person recruitment after the pandemic subsided, which prevents contemporaneous comparisons between the in-person and remote protocols. Nonetheless, results were consistent when comparing pre-pandemic enrollments to 2022-2023 enrollments (a timeframe that reflected relative normalcy[15]). Another large DPP study found favorable outcomes with a remote protocol during the pandemic, as compared to an in-person pre-pandemic protocol, controlling for individual covariates (e.g., sex, ethnicity, BMI) [18]. Our study further controlled for identification and outreach methods, and the staff who conducted outreach activities, but other unknown factors might have influenced outcomes. Therefore, future studies are needed to compare an in-person protocol to a remote protocol during the same timeframe.

In summary, compared to an in-person protocol, our remote study protocol enrolled more participants overall and from diverse, underrepresented groups in a clinical trial. The findings suggest that remote study protocols may support recruitment efforts for diabetes research trials, potentially for DPP enrollment more broadly, and appeal to more participants who could otherwise be deterred by in-person activity requirements. Efforts to help potential participants from all priority populations engage in clinical trials may lead to better clinical care and health equity.
